# Insights into Gene Transcriptional Regulation of *Kayvirus* Bacteriophages Obtained from Therapeutic Mixtures

**DOI:** 10.3390/v14030626

**Published:** 2022-03-17

**Authors:** Sara Arroyo-Moreno, Colin Buttimer, Francesca Bottacini, Nina Chanishvili, Paul Ross, Colin Hill, Aidan Coffey

**Affiliations:** 1 Department of Biological Sciences, Munster Technological University, T12 P928 Cork, Ireland; sara.arroyo-moreno@mycit.ie (S.A.-M.); francesca.bottacini@mtu.ie (F.B.); 2 APC Microbiome Ireland, University College, T12 YT20 Cork, Ireland; colin.buttimer@ucc.ie (C.B.); p.ross@ucc.ie (P.R.); c.hill@ucc.ie (C.H.); 3 George Eliava Institute of Bacteriophage, Microbiology & Virology, Tbilisi 0160, Georgia; nina.chanishvili@pha.ge; 4 School of Microbiology, University College Cork, T12 YN60 Cork, Ireland

**Keywords:** MRSA, *Staphylococcus*, *Kayvirus*, transcription, phage cocktails

## Abstract

Bacteriophages (phages) of the genus *Kayvirus* of *Staphylococcus aureus* are promising agents for therapeutic applications. In this study, we isolated *Kayvirus* phages, SAM1 and SAM2, from the *Fersisi* commercial phage cocktail (George Eliava Institute, Tbilisi, Georgia), which exhibits high sequence homology with phage K (≥94%, BLASTn). We found that phages SAM1 and SAM2 infected 95% and 86% of 21 MRSA of differing sequence types (MLST, SCCmec type) obtained from the Irish National MRSA collection, respectively. We conducted differential transcriptomic analysis by RNA-Seq on phage SAM1 during host infection, showing differential expression of its genes at different points during host infection. This analysis also allowed the identification of potentially adverse outcomes in the application of these phages to target MRSA as therapy. The interaction of phage SAM1 on the host caused the upregulation of prophage genes. Additionally, phage infection was found to cause the slight upregulation of host genes implicated in virulence factors relating to hemolysins, immune evasion, and adhesion, but also the downregulation of genes associated with enterotoxins. The findings of this study give further insights into the biology of kayviruses and their use as therapeutics.

## 1. Introduction

Phages are the most abundant biological entities in the biosphere, playing a critical role in bacterial biology, diversity, and evolution. Their global population has been estimated to be on the order of 10^31^ [1]. Lytic phages have been employed to treat bacterial infections since their discovery in the early 20th century. However, the commercialization of antibiotics from the 1940s caused phage therapy, the use of phages for therapeutic purposes, to decline in Western countries. Recent decades have seen increased research into the development of phage therapy as a viable alternative to antibiotics to eliminate multidrug-resistant pathogens.

The genus *Staphylococcus* includes several species that inhabit humans and animals, with a number of these species being opportunistic pathogens of humans. *Staphylococcus aureus* can cause infections, which manifest through various symptoms, ranging from relatively mild to life-threatening. The treatment of these conditions is becoming increasingly problematic due to the widespread dissemination of antibiotic-resistant strains, such as methicillin-resistant *Staphylococcus aureus* (MRSA) [2]. Naturally occurring virulent phages that infect and kill a wide range of *S. aureus* strains may become an alternative treatment for otherwise incurable infections caused by antibiotic-resistant staphylococci. However, when selecting phages for therapeutic applications, care must be taken to avoid those with virulence factors or genes implicated in lysogeny [3,4,5].

The *Kayvirus* genus (Twort-like) of phages of *S. aureus* are a promising agent for phage therapy due to their broad host range and high killing activity against clinical isolates of this bacterium [6,7]. These phages are often found in commercial therapeutic phage preparations [8,9,10]. They have been demonstrated to be helpful in the treatment of human infections, such as diabetic toe ulcers and musculoskeletal infections, where antibiotic therapy has failed or had limited success [11,12]. Furthermore, they have the ability to mutate and evolve to overcome bacterial defences, enabling the updating of phage therapeutics [6]. This is an advantage over traditional antibiotics, as resistance can result in their reduced antibiotic efficacy, where they can become ineffective as therapeutics. The genus *Kayvirus* belongs to the subfamily *Twortvirinae* within the family *Herelleviridae* [13]. Phages in this group are closely related and are strictly lytic [14]. Morphologically, they are characterized by an isometric head and a long contractile tail (170–220 nm). Their average genome size is about 130 kbp, featuring a pair of long direct repeats at their ends, up to several-thousand bases in length [15]. Most phages from this genus utilize the backbone of teichoic acid, a ubiquitous molecule associated with the cell wall of *Staphylococcus*, as their host cell receptor [16].

This study examines the *Kayvirus Staphylococcus* phages SAM1 and SAM2 obtained through *Fersisi* commercial phage preparation (George Eliava Institute, Tbilisi, Georgia). The George Eliava Institute developed the product approximately 15–20 years ago, based on *Pyophage*, albeit with fewer phage components. We examine the host range of two phages on a panel of MRSA representative sequence types obtained from an Irish MRSA collection from St James’s Hospital, Dublin. We also performed RNA-Seq analysis of SAM1 during the infection of its host *S. aureus* strain E1185(IV)ST12 to improve current understanding of how *Kayvirus* phages transcribe their genes during host infection but also investigate the response of the host to such infection. This analysis gives insight into potential safety concerns of using these phages on MRSA in a therapeutic context. This includes the implications of virulent phage infection on host-associated pathogenicity islands (SaPIs) or prophages, as both elements are important in the horizontal transfer of antibiotic resistance and virulence genes [17,18,19].

## 2. Results

### 2.1. Host Range of Staphylococcus Phages SAM1 and SAM2

In a previous study, we assessed the host range of *Staphylococcus* phage K on a panel of 21 Irish MRSA isolates obtained from the Irish MRSA collection at St. James’s Hospital Dublin, where phage K was found to form plaques on 43% (9/21) of the tested isolates [14]. Among the resistant strains, two were selected to isolate novel phages from *Fersisi* therapeutic phage preparation from the George Eliava Institute, namely strains E1185(IV)ST12 and E1139(IV)ST45. In the case of these two isolates, phage K showed limited infectivity on E1185(IV)ST12 (efficiency of plaquing, 1.16 × 10^−6^) and no ability to plaque on strain E1139(IV)ST45.

Phages SAM1 and SAM2 were isolated using the resistant strain E1139(IV)ST45 and E1185(IV)ST12, respectively. Host range studies for both phages were performed on 21 MRSA strains and the usual propagating host for phage K, strain DPC5246 (Table 1). All the MRSA isolates were typed based on MLST (based on seven loci) and the composition of their SCCmec element [20,21]. SAM1 was found to be capable of lysing 21 of the 22 *S. aureus* isolates. The efficiency of the plaquing (EOP) values for SAM1 were found not to be less than 1 × 10^−3^, with a maximum value of 1.2. For phage SAM2, it could form plaques on 19 of the 22 staphylococcal isolates, with EOP values ranging from 7.37 × 10^−1^ to 75.47. This phage had a low EOP on strain E1185(IV)ST12, the host for phage SAM1. Hence, there was complete coverage of the 22-strain collection with these two phages.

### 2.2. Genomic Features of Phages SAM1 and SAM2

The genome sequences obtained for phages SAM1 and SAM2 were 139,310 bp and 137,617 bp in length (excluding terminal repeats), with 212 and 211 CDSs, respectively. Four tRNA genes were found to be associated with each of their genomes, which possessed a GC content of 30%. These genomic features were similar to those reported for other phages of the *Kayvirus* group [10,14]. The CDSs were identified to encode proteins associated with transcription, DNA replication, virion structure, host lysis, and putative genes for homing endonucleases. No genes were predicted to be implicated in bacterial virulence, antibiotic resistance, or lysogeny (Supplementary Information S1, Tables S1 and S2).

A nucleotide pairwise sequence alignment based on BLASTn showed the genomes of SAM1 and SAM2 to possess 97% nucleotide sequence identity, while they shared 94% and 95% identity with phage K, respectively. A comparison of the proteomes of the phages SAM1, SAM2, and K showed that 19 ORFs were not present in all three phages. Twelve ORFs were found to be associated with phage SAM1 and SAM2, which was not found with phage K. The predicted size of their gene products ranged from 3.7 to 46.9 kDa. Apart from the ORF *SAM1_142/SAM2_141*, annotated as a uracil-DNA glycosylase, no function could be assigned to their gene products (Supplementary Information, Table S3).

### 2.3. Phage SAM1 Gene Transcription Analysis

The RNA sequencing of a single infection cycle of phage SAM1 on the *S. aureus* strain E1185(IV)ST12 at a multiplicity of infection (MOI) of 10 was performed. Under the conditions set out for this experiment, phage SAM1 possessed a latent period of 50 min. Therefore, we collected samples at 15, 35, and 45 min for analysis, with biological triplicates taken for each time-point. Unique reads that mapped to the phage or host over specified time-points ranged between 42–51% and 49–59% of the total reads, respectively (Supplementary Information S2, Figure S1). A principal component analysis (PCA) of the reads that mapped to SAM1 showed that the samples collected from the same time-point were more similar than those obtained from different times (Supplementary Information S2, Figure S2 and Table S4).

**Figure 1:**
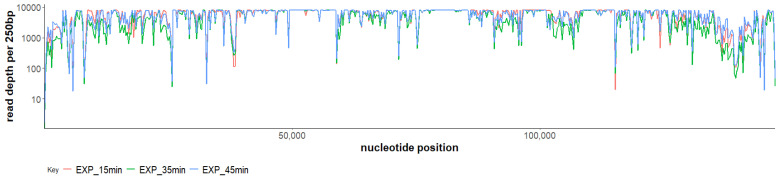
Transcription of *Staphylococcus* phage SAM1 genome during infection of *Staphylococcus aureus* at 15 min, 35 min, and 45 min using standard RNA-Seq.

**Figure 2:**
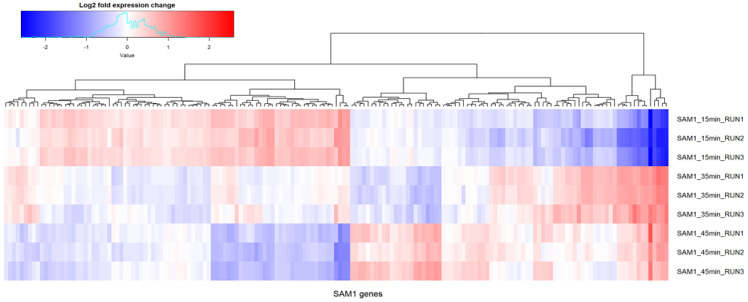
Time course transcriptional response of the genome of *Staphylococcus* phage SAM1. Values are reported as log2 fold expression change of transcripts from three independent experimental runs taken at time-points 15 min, 35 min and 45 min (15 min was set as a reference level for differential gene expression analysis); only genes with a significantly different expression between any of the time-points are shown (*p* < 0.05 in DESeq2, *n* = 169 (non-redundant genes)).

**Figure 3:**
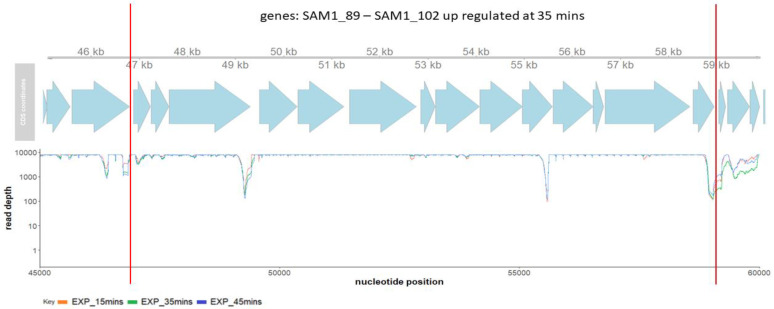
Comparison of RNA-Seq sequence depth with gene map (region spanning bases 45,000 to 60,000) of *Staphylococcus* phage SAM1. The gene map comprises of arrows representing genes, their length and location, with the direction of the arrow indicating direction transcription. It can be observed that areas of low sequence coverage often correlate with the gene cluster boundary (red lines) that are up regulated at similar time points during phage infection of the host.

Gene expression was found to have occurred throughout the genome of phage SAM1 among the time-points selected for the RNA-Seq analysis. No region on the genome of this phage was found to have an absence of aligned reads (Figure 1). A previous transcriptional study of kayvirus phage vB_SauM-515A1 produced similar findings [15]. However, locations on the genome of SAM1 with low read depth were identified between genes *SAM_75* and *SAM_76*, and between *SAM1_213* and *SAM1_214*. The former region’s low GC content (20%) indicates that it may be the proximal locus for the origin of replication for the SAM1 genome [22]. However, a differential expression analysis conducted using DESeq2 showed significantly differentially expressed genes (*p* < 0.05) of the phage throughout infection (Figure 2). Differences in gene expression were typically observed in genes belonging to the same transcriptional unit, encoding proteins of related function and, possibly, co-transcribed. Notably, a sharp decrease in sequence coverage often matched the predicted boundaries of these transcriptional units, which were typically situated between the boundaries of the ORFs (Figure 3). These low-coverage regions likely correlated with the common start and endpoints of the mRNA transcripts.

A comparison of the transcription at 15 min with other time-points (35 and 45 min) of phage infection showed a preferential expression for transcriptional units with genes implicated in nucleotide metabolism and DNA replication, repair, or recombination (as observed for transcriptional units spanning from SAM*1_58–71* to SAM*_124–154* and *SAM_158–162*). This observation was also made for three of the four tRNA genes of the phage, which would make these tRNAs available throughout infection. A similar comparison showed that transcriptional units with genes implicated in virion structure (*SAM_076–123* and *SAM_155–157*) and cell lysis, such as endolysin (*SAM_072*) and holin (*SAM_073*), were most upregulated at 35 min compared to 15 or 45 min.

The most notable differential gene expression was found in gene *SAM1_045*, which was highly upregulated at 45 min compared to its expression at 15 min. Its gene product is a putative cell-wall binding domain (LysM, HHpred: probability > 95%). This differential expression suggests that the gene product likely plays a role in host cell lysis. Examples of multimeric endolysins include PlyC of *Streptococcus* phage C_1_, where the cell-wall binding domain and peptidoglycan degradation are encoded on separate genes [23]. However, *SAM_072* encodes an endolysin, where a cell-wall binding domain (SH3, IPR003646) is associated with a peptidoglycan domain (CHAP, IPR007921). Thus, the putative role of the gene product *SAM1_045* at the late stage of infection is unclear.

A complete list of SAM1 genes that showed differential expression (*p* < 0.05, DESeq2) during host infection are presented in Supplementary Information S1, Table S5.

*Kayvirus* phages do not possess genes for RNA polymerase and therefore exploit those of the host bacterium. An examination of genes upregulated at 15 and 35 min showed the presence of putative promoters that were highly similar, resembling the canonical σ70 promoter with a consensus sequence -35 motif (TTGACA) and -10 motif (TATAAT). The genes upregulated at 45 min possessed similar promoters, but with a -10 motif that had a more divergent consensus sequence (Supplementary information S1, Table S5 and Figures S3–S5). These predicted promoters of SAM1 were similar to those previously reported among other kayviruses [15]. Additionally, one of the transcriptional units upregulated at 15 min terminated with a gene encoding an RNA polymerase sigma factor (SAM1_154, HHpred: probability > 99%), which was upregulated at this time-point but downregulated at subsequent time-points. It is expected that this factor may play a role in the temporal gene expression of this phage, as such elements cause the alteration of promoter sequences recognized by RNA polymerase. In the case of SAM1, possibly causing the host RNA polymerase to recognize promotors with the alternative -10 motif identified among the genes upregulated at the later stages of phage infection.

### 2.4. Host Gene Transcription Analysis during Phage SAM1 Infection

After sequencing with a combination of long and short reads, the genome of *S. aureus* strain E1185(IV)ST12 returned as a complete circular chromosome of 2,782,853 bp containing 2704 coding sequences (CDSs) and 59 tRNA genes. The isolate also possesses a plasmid 26,732 bp in size with 27 CDSs. Its genome was observed to contain the SCCmec_type_IVc(2B) element with the strain associated with MLST type ST12. RNAseq analysis showed alternation of the transcriptional regulation of genes of the host during phage SAM1 infection over selected time-points. A complete list of genes that showed differential expression (*p* < 0.05, DESeq2) during host infection are presented in Supplementary Information S1, Table S6.

A comparison of host gene expression at 15 and 45 min during phage infection (Table 2) shows slight upregulation of the virulence factor genes implicated in the formation of hemolysins (*hyl*, *hlgC*). This was also detected for gene-encoding proteins with a role in subverting the human immune system, such as staphylococcal protein A (*sbi*) and complement inhibitor (*scn*). Similar observations were made for the products implicated in adherence of the bacterium to surfaces, such as extracellular adherence protein (*eap*) and fibronectin-binding protein A (*fnbA*). The virulence-factor genes in which slight downregulation was observed were those for staphylococcal enterotoxins (*sel26, selZ*). Transcriptional upregulation was also detected in a prophage (spanning ORFs *LUU_550–797)* located on the genome of the *S. aureus strain* E1185(IV)ST12 belonging to the genus *Phietavirus*. Similar studies with *Pseudomonas aeruginosa* have also shown the transcriptional upregulation of prophage elements during virulent phage infection [24].

## 3. Discussion

Numerous studies report the inefficacy of phage K against several MRSA strains [9,14]. In the present study, two phages belonging to the same genus as phage K were isolated from the George Eliava Institute’s *Fersisi* commercial phage mix, namely SAM1 and SAM2. These two phages present a much broader host range against MRSA strains from the National Reference Collection in comparison with phage K, which effectively only lysed nine strains out of twenty-one [14]. This strain collection was used because the differing sensitivity/resistance profiles that can be observed with *Kayvirus* phages can inform us as to which phages have the best potential for use in therapeutic mixes that might be adapted to eliminate specific sequence types from specific MRSA infections in Ireland. The strains selected for the isolation and propagation of phages SAM1 and SAM2, namely *S. aureus* E1185(IV)ST12 and *S. aureus* E1139(IV)ST45, were previously shown to have poor sensitivity to phage K and, indeed, two other phages from the genus *Kayvirus* [14]. As phage K can only infect *S. aureus* E1185(IV)ST12 at an extremely low EOP and cannot produce plaques on *S. aureus* strain E1139(IV)ST45, it is interesting to observe how minor differences between the SAM1/SAM2 genomes and that of phage K might play a role in phage virulence. The SAM1 and SAM2 phage genomes shared 94% and 95% nucleotide sequence identity (BLASTn), respectively, with phage K. More significant steps now need to be taken to understand the factors these differences contribute to *the Kayvirus* host range. This knowledge would enable enhanced phage therapeutics based on these phages. However, this study indicates the potential of key individual component phages from the *Fersisi* commercial phage mix for the elimination of Irish MRSA strains.

A transcriptional analysis of *Kayvirus* phage SAM1 showed the temporal regulation of gene expression as observed for other myoviruses, such as phage PAK_P3 of the genus *Nankokuvirus*, infecting *Pseudomonas* [25]. The genes of this phage can be primarily categorized as those upregulated during the early, middle, or late stage of infection. Early genes encode hypothetical proteins of unknown function, middle genes focus mainly on nucleic acid metabolism, and late genes are for virion structure. Under the conditions set out by the one-step growth curve of *Staphylococcus* phage SAM2 on host *S. aureus* strain E1185(IV)ST12 in this study, a latent period of 50 min was determined with three-time-points being selected for RNA-Seq analysis. In the case of *Staphylococcus* phage SAM1, the genes upregulated in early infection (15mins) were primarily those involved in nucleotide metabolism and functions related to DNA replication and repair. The genes upregulated toward the middle (35 min)/late (45 min) stage of infection were those implicated in virion structure and host cell lysis. These changes in gene expression levels possibly related to the gene *SAM1_154* encoding an RNA polymerase sigma factor, upregulated at the 15 min time-point and homologous with that of *Bacillus* phage SPO1 gene *gp34*, implicated in the late gene expression of that phage [26].

During the infection of *S. aureus* E1185(IV)ST12 by phage SAM1, genes of a prophage element were shown to be upregulated at 45 min, indicating that this prophage was exiting the host chromosome and entering the lytic cycle. This observation draws attention to the concern that such elements are known to be agents of horizontal gene transfer [17,18]. Additionally, they can harbor genes that influence host fitness (i.e., pathogenicity, prophage immunity, biofilm formation, and stress response), potentially promoting their spread among sensitive strains of *S. aureus* [27]. Further investigation is merited to gauge the impact of *Kayvirus* lytic infection on indigenous prophage induction in *S. aureus*. It is worth mentioning that prophage induction can also occur during the application of antibiotics [28]. The analysis in this study also showed the slight upregulation of host genes implicated in virulence factors relating to hemolysins, immune evasion, and adhesion during phage infection. However, it must be stated that the application of these phages in the treatment of severe staphylococcus infections has been shown to be safe: no adverse reactions were reported among thirteen tested patients [29]. Thus, the observations in this study should not be a barrier to the use of kayviruses as therapeutics.

## 4. Materials and Methods

### 4.1. Bacterial Strains, Phage, and Growth Requirements

Phages SAM1 and SAM2 were isolated from the *Fersisi* commercial phage cocktail from the George Eliava Institute of Bacteriophage, Microbiology and Virology, Tbilisi, Georgia. The MRSA strains utilized for host range studies were predominantly the Irish National MRSA Reference collection, except for DPC5246, the routine propagation of phage K in our laboratory [14,30,31]. The MRSA strains used for the isolation and routine propagation of phages SAM1 and SAM2 were *S. aureus* E1185(IV)ST12 and *S. aureus* E1139(IV)ST45, respectively.

*S. aureus* isolates were routinely cultured in brain–heart infusion broth (BHI; Sigma-Aldrich, St. Louis, MO, USA) at 37 °C or on BHI plates containing 1.5% (*w*/*v*) bacteriological agar (Sigma-Aldrich). All strains were stocked in BHI containing 40% (*v*/*v*) glycerol and stored at −80 °C. Phage propagation was performed using plaque assay plating technique, propagation with BHI broth, and plate propagation with SM buffer [32].

### 4.2. Phage Host Range

Host range assay was performed for phages SAM1 and SAM2 using the plaque assay plating technique. This was performed in triplicate for three independent experiments. The efficiency of plaquing (EOP) was determined by dividing the phage titre on each test strain by the phage titre of the reference strain (*S. aureus* E1185(IV)ST12, in the case of phage SAM1, and *S. aureus* E1139(IV)ST45 for SAM2) [33].

### 4.3. DNA Extraction, Sequencing, and Genome Assembly

Phage DNA extraction was performed on high-titre phage lysates, as described by Ajuebor et al. in 2018 [14]. Briefly, phage samples were initially treated with MgCl_2_, followed by pre-treatment with DNase and RNase for 60 min at 37 °C. Next, subsequent treatments with SDS, EDTA, and proteinase K with further incubation for 60min at 55 °C were performed. DNA extractions were then performed with phenol/chloroform/isoamyl alcohol (25:24:1 *v*/*v*/*v*) and chloroform/isoamyl alcohol (24:1 *v*/*v*). DNA precipitation was achieved using sodium acetate and 95% (*v*/*v*) ethanol. DNA quality and quantity were estimated using a Nanodrop (ND-1000) and visualized in a 1% agarose gel electrophoresis.

Genomic sequencing was outsourced to genomic DNA MicrobesNG, Birmingham University, England, United Kingdom. For Illumina DNA sequencing, genomic DNA was isolated using the QIAamp DNA Mini Kit (Qiagen, Hilden, Germany). DNA was quantified in triplicates with a Quantit dsDNA HS assay in an Eppendorf AF2200 plate-reader. Genomic DNA libraries were prepared using Nextera XT Library Prep Kit (Illumina, San Diego, CA, USA) following the manufacturer’s protocol with the following modifications: two nanograms of DNA instead of one were used as input, and PCR elongation times was increased to 1 min from 30 s. DNA quantification and library preparation were carried out on a Hamilton Microlab STAR automated liquid handling system. Pooled libraries were quantified using the Kapa Biosystems Library Quantification Kit (Roche, Basel, Switzerland) for Illumina on a Roche Lightcycler 96 qPCR machine. Libraries were sequenced on an Illumina instrument using 250 bp paired-end protocol.

For Nanopore sequencing, broth cultures of each isolate were then pelleted and resuspended in the tube with cryopreservative (Microbank, Pro-Lab Diagnostics UK, Wirral, UK). Approximately 2 × 10^9^ cells were used for high-molecular-weight (HMW) DNA extraction using Nanobind CCB Big DNA Kit (Circulomics, Baltimore, MA, USA). DNA was quantified with the Qubit dsDNA HS assay in an Invitrogen Qubit 3.0 (Thermo Fisher Scientific, Waltham, MA, USA). Long-read genomic DNA libraries were prepared with Oxford Nanopore SQK-RBK004 kit with Native Barcoding EXP-NBD104/114 (Oxford Nanopore Technologies, Oxford, UK) using 500 ng of HMW DNA. Barcoded samples were pooled together into a single sequencing library and loaded in a FLO-MIN106 (R.9.4) flow cell in a GridION (Oxford Nanopore Technologies).

Illumina reads were adapter-trimmed using Trimmomatic (v0.30) with a sliding window quality cut-off of Q15 [34]. For phage genomes (SAM1 and SAM2), assembly was performed on samples using SPAdes (v3.7) [35]. For bacterial host (*S. aureus* strain E1185(IV)ST12), genome assembly was conducted with Unicycler (v0.4.8) using short and long reads obtained from Illumina and Nanopore sequencing, respectively [36].

### 4.4. Genome Annotation and Comparison

RAST was employed to predict ORFs of sequenced phages, while ARAGORN was used to predict tRNA genes [37,38]. Further annotation was derived from BLASTP using the non-redundant nr/nt database [39], along with HHpred [40] and InterProscan [41]. Lipoproteins were identified using LipoP [42]. TMHMM was used to identify transmembrane domains [43]. Identification of *Kayvirus* virions structural proteins was determined by comparison with those found for *Staphlococus* phage ISP [44]. The proteomes of phages K (GenBank accession number: NC_005880), SAM1 and SAM2 were compared with Proteinortho using default parameters [45]. Potential promoters were identified by submitting up-stream sequences (250 bp) of predicted genes to multiple em for motif elicitation (MEME) [46]. The genome of *Staphylococcus aureus* strain E1185(IV)ST12 was annotated with PGAP [47]. Bacterial genomes (only E1185(IV)ST12 was made available for this manuscript) were analyzed with SCCmecFinder to find SCCmec element type [20] and MLST using PubMLST [48]. Virulence genes of the host were identified using the Virulence Factor Database (January 2022) [49].

### 4.5. RNA-seq Analysis of Phage SAM2

A one-step growth curve was created. *S. aureus* strain E1185(IV)ST12 was grown to an OD600 of 0.6 (corresponding to approximately 4.8 × 10^8^ CFU/mL), followed by centrifugation of 2 mL volume in a microfuge to pellet bacteria. The bacterial pellets were resuspended in 1 mL of phage lysate to yield an approximate multiplicity of infection MOI of 10, following incubation at 37 °C for 1 min and subsequent centrifugation to eliminate unbound phages. Bacterial pellets with bound phages were resuspended in a total volume of 10 mL of BHI broth incubated aerobically in a water bath at 37 °C with agitation at 60 rpm. At time-points of 15, 30 and 45 min, 1.8 mL samples were taken into 1/10 volume of stop solution (10% phenol in 100% ethanol) and immediately placed on ice. These tubes were then centrifuged for 1 min at 16,000× *g* at room temperature. Pellets were resuspended in 800 μL of Tri Reagent (Sigma-Aldrich) and transferred to fresh tubes containing acid-washed beads. These tubes are placed in a ribolyser (MagNA Lyser, Roche), with beads subsequently removed by centrifugation. Supernatants were subject to total RNA extraction using a Direct-zol RNA miniprep kit (Zymo Research, Irvine, CA, USA, as per manufacturer instructions). RNA samples were subject to rRNA depletion before library creation. Paired-end sequencing was performed using an IlluminaHiSeq sequencer (Illumina Inc., San Diego, CA, USA).

RNA-seq reads were mapped to genomes (genomic features model GTF file5) of *Staphylococcus* phage SAM1 and that of host S. *aureus* strain E1185(IV)ST12 through Bowtie2 (v2.3.4.1) [50]. The alignment SAM files were further processed using Samtools (v1.7) to obtain BAM files necessary to obtain matrices with read counts per gene (normalized by gene length) [51]. Differential gene expression (DGE) analysis was performed using the R environment and the DESeq2 package available as part of the Bioconductor release [52]. As a pre-processing step, rows with zero counts (unmapped genes) were discarded from the count matrices. Differential expression analysis was performed on the count matrices using the DESeq function in DESeq2.

### 4.6. Accession Numbers for Sequence Data

Genome sequences of *Stapholococus* phages SAM1 and SAM2 were deposited under GenBank accession numbers MT338525 and MT226657, respectively. The genome and plasmid of *S. aureus* strain E1185(IV)ST12 were deposited under Genbank accession numbers CP089586 and CP089587, respectively. Sequence reads from the transcriptional analysis of phage SAM1 infection of *S. aureus* strain E1185(IV)ST12 were submitted to GEO under accession number GSE192733.

## Figures and Tables

**Table 1:** Host ranges of staphylococcal phages SAM1 and SAM2 on 22 *Staphylococcus aureus* strains, comprising of 21 MRSAs of differing sequence types obtained from the Irish National MRSA collection and DPC5246, the propagating host of phage K. The table documents the efficiency of plaquing (EOP) values.

*S. aureus* Strain	SCCmec Type	MLST	Bacteriophages	
			SAM1	SAM2
			EOP ± SD	EOP ± SD
DPC5246	Not detected	71	1.2 ± 0.4	3.72 ± 0.48
0.0066(IIv)ST239	II(2A) **	36	5.1 × 10^−1^ ± 0.1	4.37 ± 1.8
0.1206(IV)ST250	IVc(2B)	12	5.74 × 10^−1^ ± 0.4	no plaques
0.1239(III)ST239	III(3A)	239	1.4 × 10^−1^ ± 0.05	6.6 ± 2.46
0.1345(II)ST5	VIII(4A)	8	2.23 × 10^−1^ ±0.1	1.96 ± 0.51
0073(III)ST239	III(3A)	239	1.21 × 10^−1^ ± 0.1	no plaques
0104(III)ST239	III(3A)	239	9.65 × 10^−2^ ± 0.01	3.5 ± 1.19
0220(II)ST5	IV(2B) **	8	2.22 × 10^−2^ ± 0.003	14.68 ± 4.31
0242(IV)ST30	II(2A)	496	1.95 × 10^−1^ ± 0.17	9.35 ± 3.15
0308(IA)ST247	I(1B)	247	3.41 × 10^−1^ ± 0.26	11.85 ± 3.24
3045(IIv)ST8	II(2A)	8	1.94 × 10^−1^ ± 0.14	4.54 ± 2.13
3144(IIv)ST8	II(2A)	8	2,17 × 10^−1^ ± 0.22	10.46 ± 5.18
3488(vv)ST8	IV(2B) **	8	6.72 × 10^−2^ ± 0.01	11.11 ± 4.35
3581(IA)ST247	VIII(4A) **	8	1 × 10^−1^ ± 0.01	5.51 ± 1.25
3594(II)ST36	III(3A)	239	5.77 × 10^−1^ ± 0.5	75.47 ± 2.32
3596(IIv)ST8	VIII(4A) **	8	2.29 × 10^−1^ ± 0.05	28.89 ± 16.48
E1038(IIv)ST8	II(2A)	8	9.7 × 10^−1^ ± 0.97	13.47 ± 3.47
E1139(IV)ST45	IVa(2B)	45	no plaques	1 *
E1174(IV)ST22	IV(2B)	22	1.4 × 10^−1^ ± 0.01	7.37 × 10^−1^
E1185(IV)ST12	IVc	12	1 *	3.5 × 10^−4^ ± 3.3 × 10^−4^
E1202(II)ST496	VIII(4A) **	8	3.59 × 10^−1^ ± 0.2	20.83 ± 3.24
M03/0073(III)ST239	III(3A)	239	1.32 ± 0.7	16.54 ± 0.52
0104(III)ST239	III(3A)	239	1.2 ± 0.4	3.72 ± 0.48
0220(II)ST5	IV(2B) **	8	5.1 × 10^−1^ ± 0.1	4.37 ± 1.8
0242(IV)ST30	II(2A)	496	5.74 × 10^−1^ ± 0.4	no plaques

* Host strain of phage. ** SCCmec type: two possible types suggested by SCCmecFinder with a hit of greatest coverage selected.

**Table 2:** List of virulence-factor genes of *S. aureus* strain E1185(IV)ST12 that experience up/downregulation during infection of *Staphlococcus* phage SAM1 (*p* < 0.05, DESeq2).

Virulence Factors (Locus_tag, Product, Gene)	log2 Fold Change		
	15 min	35 min	45 min
LUU82_11680—bi-component gamma-hemolysin HlgCB subunit C—*hlgC*	−0.11173	−0.0293	0.141026
LUU82_04645—alpha-hemolysin—*hyl*	−0.15039	−0.04266	0.193054
LUU82_09700—staphylococcal protein A—*spa*	−0.26288	−0.0223	0.285182
LUU82_09135—complement inhibitor SCIN—*scn*	−0.18095	−0.16701	0.347955
LUU82_04665—complement convertase inhibitor—*efb*	−0.44123	−0.06633	−0.507566
LUU82_00160—extracellular adherence protein Eap/Map—*eap*	−0.18624	−0.03037	0.216609
LUU82_11260—fibronectin-binding protein FnbA—*fnbA*	−0.43413	−0.09529	0.529417
LUU82_02360—staphylococcal enterotoxin type—*sel26*	0.415231	−0.08836	−0.32687
LUU82_09885—staphylococcal enterotoxin type Z—*selZ*	0.413472	−0.08253	−0.33094

## Supplementary Materials

The following supporting information can be downloaded at: https://www.mdpi.com/article/10.3390/v14030626/s1. Table S1. Annotation of the Staphylococcal phage vB_SauM_SAM1 genome. Table S2. Annotation of the Staphylococcal phage vB_SauM_SAM1 genome. Table S3. Genes of Staphylococcus phages SAM1, SAM2 and K fall into the same orthologous groups. Table S4. Filtered differential gene expression results of Staphylococcus phage SAM1 infection of Staphylococcus aureus strain E1185(IV)ST12. Table S5. Predicted promoter sequences of Staphylococcus phage SAM1. Table S6. Filtered differential gene expression results of Staphylococcus aureus strain E1185(IV)ST12 during infection by Staphylococcus phage SAM1. Figure S1. Percentage of unique reads mapping to the genomes of Staphylococcus phage SAM1 and S. aureus strain E1185(IV)ST12. Figure S2. Principal component analysis graph presenting the correlation between all the samples used in this study concerning reads mapped to the genome of Staphylococcus phage SAM1. Figure S3. Consensus sequence logos for a putative promotor of Staphylococcus phage SAM1 among genes up regulated at 15 min. Figure S4. Consensus sequence logos for a putative promotor of Staphylococcus phage SAM1 among genes up regulated at 35 min. Figure S5. Consensus sequence logos for a putative promotor of Staphylococcus phage SAM1 among genes up regulated at 45 min.
