# Supplementary material for: Insights into Gene Transcriptional Regulation of Kayvirus Bacteriophages Obtained from Therapeutic Mixtures
Source: Viruses. 2022 Mar 17;14(3):626. doi: 10.3390/v14030626 (PMC8952766; doi:10.3390/v14030626)
Supplement: Supplementary file 1 [file viruses-14-00626-s001.zip › Supplementary_information S2 - FINAL.pdf]

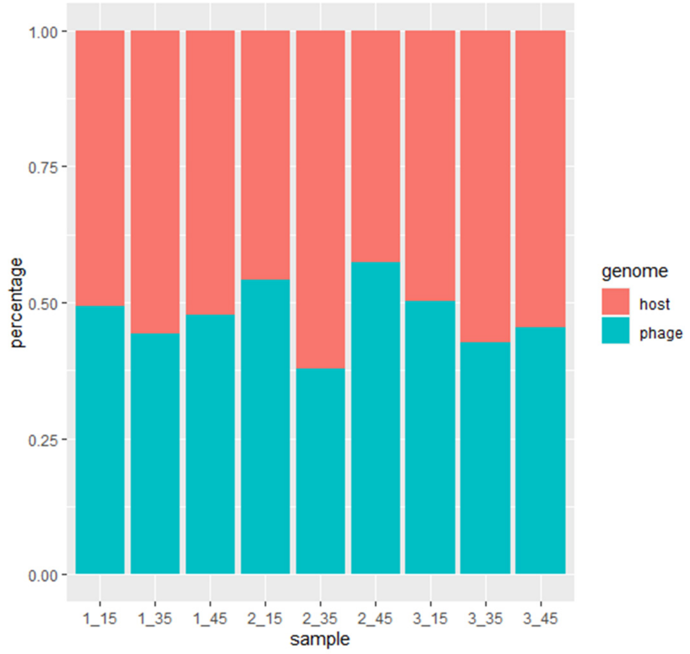

Figure S1. Percentage of unique reads mapping to the genomes of Staphylococcus phage SAM1 and *S. aureus* strain E1185(IV)ST12.

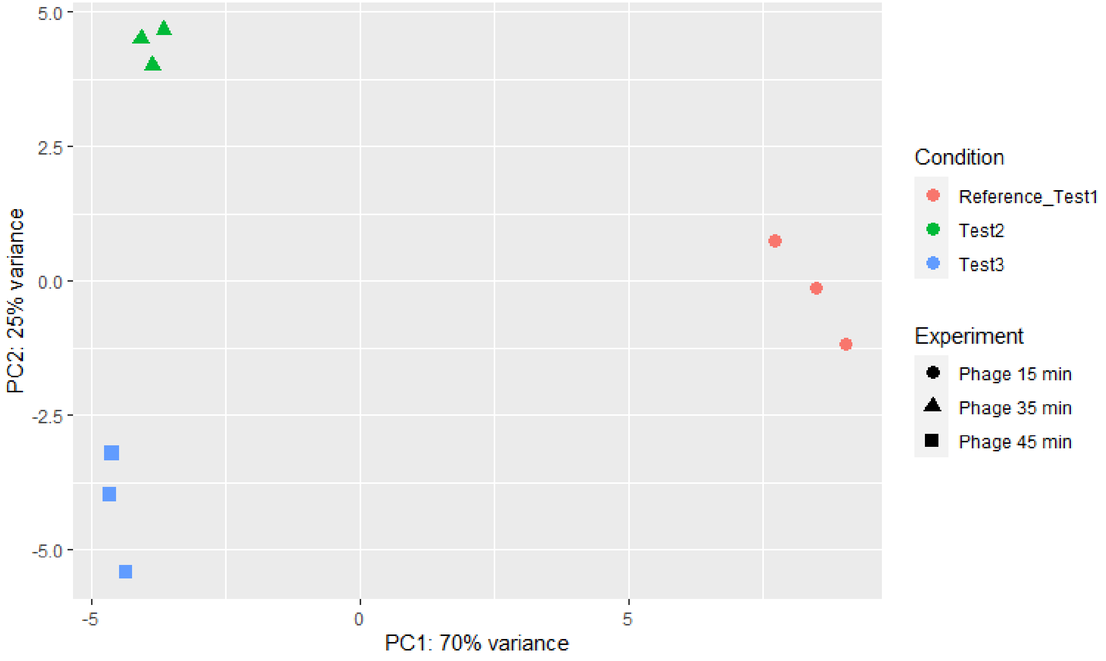

Figure S2. Principal component analysis (PCA) graph presenting the correlation between all the samples used in this study in relation to reads mapped to the genome of *Staphylococcus* phage SAM1

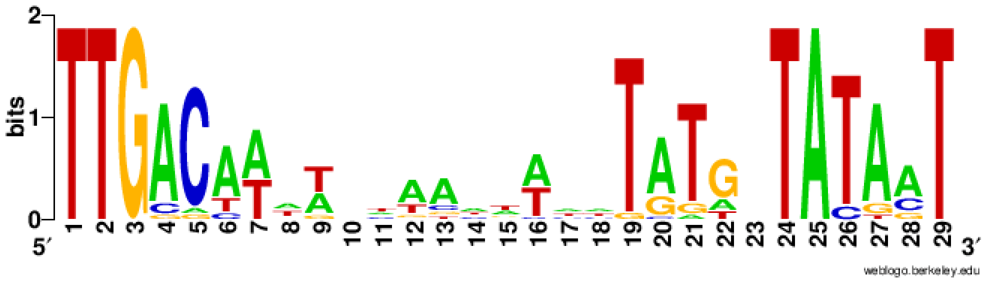

Figure S3. Consensus sequence logos for a putative promotor of *Staphylococcus* phage SAM1 among genes up regulated at 15 mins

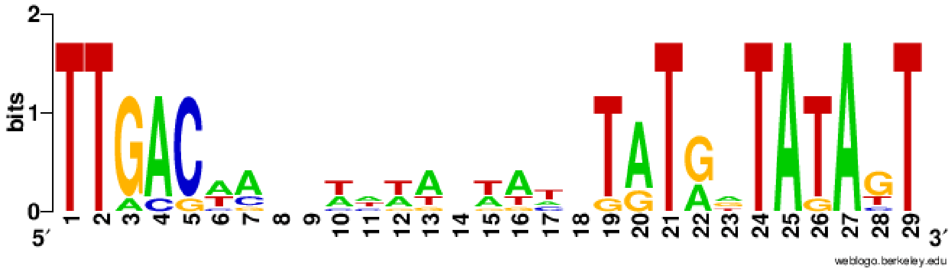

Figure S4. Consensus sequence logos for a putative promotor of *Staphylococcus* phage SAM1 among genes up regulated at 35 mins

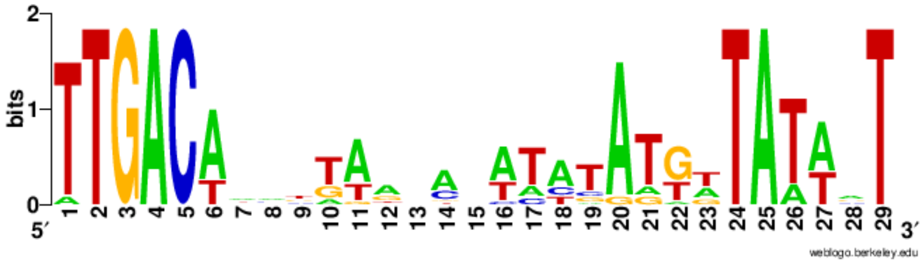

Figure S5. Consensus sequence logos for a putative promotor of Staphylococcus phage SAM1 among genes up regulated at 45 mins
